# Antibiotic Sensitivity Pattern of Common Microorganisms Isolated From Surgical Site Infections (SSIs) After Abdominal Surgeries at Ayub Teaching Hospital in Abbottabad, Pakistan

**DOI:** 10.7759/cureus.98063

**Published:** 2025-11-29

**Authors:** Hadayat Ullah, Saeed Ahmad, Bilal Khalid, Hina Javed, Ayesha Naseem, Imad Alam, Muhammad Farooq, Zeeshan Mahmood Alam, Farhan Shahzad

**Affiliations:** 1 Surgery, Ayub Teaching Hospital, Abbottabad, PAK; 2 General Surgery, Ayub Teaching Hospital, Abbottabad, PAK; 3 Surgery, Jinnah Postgraduate Medical Centre, Karachi, PAK; 4 Medicine, Khyber Medical College, Peshawar, PAK; 5 Surgery, Nowshera Medical College, Nowshera, PAK; 6 Orthopedic Surgery, Khyber Teaching Hospital, Peshawar, PAK; 7 Emergency Medicine, University Hospital Kerry, Tralee, IRL; 8 Internal Medicine, Saidu Teaching Hospital, Swat, Saidu Sharif, PAK

**Keywords:** abdominal surgeries, antibiotics, antibiotic susceptibility, surgical site infection, surveillance

## Abstract

Background

Surgical site infections (SSIs) are among the most frequent complications of surgical procedures globally and a major contributor to surgical morbidity, posing a huge burden on patients and health systems. To effectively treat SSI, the determination of sensitivity patterns is crucial to reduce the health burden and curb microbial resistance.

Objective

This study aimed to identify the most common microorganisms that cause infection at the surgical site after abdominal surgery and their antibiotic sensitivity trend.

Methods

The Department of Surgery at Ayub Teaching Hospital in Abbottabad, Pakistan, conducted this prospective observational study from March to August 2025. Through consecutive sampling, 89 patients were included in the study. IBM SPSS Statistics for Windows, Version 27.0 (IBM Corp., Armonk, New York, United States), was used for analyzing the data, and the results are presented in frequency tables and graphs.

Results

In 89 patients, the mean age was 39.3±20.9 years, 41.6% had comorbidities, and 67.4% underwent emergency surgery. Gram-negative bacteria predominated, with *Klebsiella pneumoniae* (8.9%; emergency: 7/60, 11.7%; elective: 3/29, 10.3%) and *Pseudomonas aeruginosa* (11.1%; emergency: 5/60, 8.3%; elective: 3/29, 10.3%) being the next most common organisms isolated after *Escherichia coli* (55.6%; emergency: 36/60, 60%; elective: 14/29, 48.3%). The most effective medications were still carbapenems (48.3%) and linezolid (57.3%). However, considerably high resistance was observed in penicillin (50.6%), cephalosporins (59.6%), and fluoroquinolones (61.6%).

Conclusion

The majority of the causes of SSIs were gram-negative bacteria, especially *E. coli*, which exhibit significant resistance to commonly used antibiotics. The ongoing efficacy of carbapenems and linezolid highlights the necessity of rigorous infection control and local antibiogram-guided therapies.

## Introduction

A surgical site infection (SSI) is an infection at the site of an incision, whether superficial or involving the deep tissue, that occurs within 30-90 days of surgery, depending upon the type of surgery [1,2]. Healthcare infections (HAIs) are among the major causes of morbidity and healthcare costs worldwide [2,3]. Globally, they are among the most important causes of morbidity, with SSIs ranking second in frequency after urinary tract infections [3]. This rate in Pakistan is alarming. The available literature ranges from 9% to 33% for different surgical specialties and institutions, a figure that is markedly higher than what is reported in developed countries [4]. 

Several factors are associated with an elevated risk of SSI, including patient-specific variables such as immunosuppression, extremes of ages (neonates or geriatrics), malnutrition, obesity, diabetes mellitus, immunosuppression (due to human immunodeficiency virus (HIV), malignancy, chemotherapy, or corticosteroid use), smoking, and chronic conditions such as renal or hepatic insufficiency [5]. Surgical risk factors include lengthy operations, contamination, poor sterilization, and incorrect instrument handling. Furthermore, physiological factors such as hypoxia, postoperative hyperglycemia, and multiple traumas enhance the risk of SSI [6]. Other independent risk factors, such as pathogen virulence, long hospital stay, and prolonged antibiotic use, increase the risk of multidrug-resistant infections, which further complicate the treatment of SSI [7]. 

The 68th United Nations General Assembly meeting declared SSI a global health threat. This has increased sharply in recent decades. Drug-resistant pathogens that are resistant to several antibiotics, including tetracyclines, cephalosporins, ampicillin, penicillin, and methicillin, such as methicillin-resistant *Staphylococcus aureus*, *Enterococcus *species, *Pseudomonas aeruginosa*, *Klebsiella pneumoniae*, and *Escherichia coli*, currently represent a serious threat [6-8]. However, evidence suggests that with best practices, 40-60% can be prevented [9]. Despite improvement in surgical techniques, infection control measures, antibiotic prophylaxis, and sterilization, SSIs remain one of the most frequent infections linked to healthcare, especially in countries with low and moderate incomes [9,10]. Between 2000 and 2015, the use of antibiotics rose by about 40% globally, with developing countries accounting for the majority of this growth [6-13]. This increase accelerated the growth of antibiotic resistance and the corresponding morbidity, mortality, and expenses [14,15]. One of the most important aspects of hospital antibiotic use is preventing SSI, which is the second most prevalent HAI and increases expenditure, hospital stay, and antibiotic consumption [6-7,16]. While improper timing and prolonged use of antibiotics increase the risk of developing resistance, kidney damage, and *Clostridium difficile* infection, evidence suggests that timely and appropriate antibiotic prophylaxis is effective in preventing SSIs [12-17]. Antimicrobial resistance has severe implications, particularly in developing countries such as Pakistan. Antibiotics' use, together with inadequate methods of infection control, further leads to the development of resistant conditions, especially in congested hospitals, with the lack of funding for thorough antimicrobial stewardship initiatives [18].

In light of this, current local surveillance data are essential for logical antibiotic prescription and infection prevention efforts. Therefore, it is crucial to regularly evaluate bacterial isolates and their susceptibility patterns to establish stewardship policies that are specific to each institution's requirements, guide empirical therapy, and improve prophylactic regimens [19]. Ayub Teaching Hospital, Abbottabad, is a major tertiary care referral center in Northern Pakistan. Although SSIs are clinically significant in abdominal surgery, recent data about the causative organisms and their antibiotic resistance profiles are limited. This study was therefore conducted to identify the bacteriological spectrum and antibiotic susceptibility patterns of organisms isolated from SSIs after abdominal surgery at Ayub Teaching Hospital.

## Materials and methods

Study design, setting, and timeline

This is a prospective observational study conducted at the Department of Surgery of Ayub Teaching Hospital in Abbottabad, Pakistan, from March to August 2025, after obtaining approval from the Ayub Medical Teaching Institution Abbottabad Institutional Medical and Ethics Review Committee (approval number: EA-2025/055). Written informed consent was obtained from all participants, and the data they submitted were kept confidential.

Inclusion and exclusion criteria

The participants of the study were all patients during the study period, aged >18 years, who underwent abdominal surgery and showed signs of an SSI within 30 days following the procedure (according to the Centers for Disease Control and Prevention (CDC) criteria: purulent discharge, redness, swelling, pain, or systemic signs of infection). The surgical techniques used in surgery, both elective and emergency, included laparotomy, appendectomy, cholecystectomy, and hernia repair. In contrast, patients who declined to participate in the study, died within 48 hours of arrival, or had an initial diagnosis of SSIs were excluded. 

Sample size

A total of 89 patients were included based on consecutive sampling.

Data collection

A self-generated proforma was used for data collection, containing information such as age, sex, comorbidities (including diabetes and hypertension), type of surgery, antibiotic used, SSI diagnosed days after surgery, organism isolation, and antibiotic sensitivity.

Specimen collection

Wound swabs and pus aspirates from patients who presented with signs of SSIs within 30 days of surgery were collected by a senior surgical resident or a qualified surgeon and sent to the laboratory under strict aseptic conditions.

Transport

Specimens were immediately transferred to a sterile container and sent to the laboratory.

Culture and identification

Standard culture techniques were applied with bacterial identification and biochemical testing.

Antibiotic susceptibility testing

Antibiotics tested included penicillins, cephalosporins, fluoroquinolones, aminoglycosides, carbapenems, macrolides, linezolid, vancomycin, and β-lactam/β-lactamase inhibitor combinations.

Furthermore, safety-focused antibiotic stewardship and SSI prevention policy of our institute clearly advises to provide prophylaxis only when it is truly needed, using options like Augmentin, ceftriaxone, metronidazole, or piperacillin-tazobactam, and always within an hour of the incision before surgery, and these drugs are stopped after 24 hours to avoid unnecessary exposure. Antibiotics are tailored according to culture results, and antibiotics such as carbapenems, vancomycin, linezolid, or colistin are reserved for multidrug-resistant cases and require formal approval to ensure the safety of patient health.

SSI diagnosis

SSI was diagnosed according to the CDC criteria, based on purulent discharge, localized pain, redness, swelling, or systemic signs of infection, and confirmed by microbiological culture and antimicrobial sensitivity testing. Descriptive statistics were used to demonstrate antimicrobial susceptibility patterns.

Data analysis

Data was analyzed using IBM SPSS Statistics for Windows, Version 27.0 (IBM Corp., Armonk, New York, United States). The qualitative data were presented as percentages and frequencies, while quantitative data were presented as means, medians, and standard deviations. For categorical data, e.g., gender and type of surgery, the chi-squared test was applied, while for continuous variables such as age and hospital stay, the independent t-test was applied. Statistical significance was set at p<0.05.

## Results

During the study period, 247 patients underwent abdominal surgery, resulting in 89 SSIs. Of the 89 patients, 47 (52.8%) were female, and 42 (47.2%) were male (p=0.063) with no statistically significant difference in gender distribution. A significantly high level of patients underwent emergency surgery (n=60 (67.4%)) as compared to elective surgeries (n=29 (32.6%)). The chi-squared test was applied (χ²=5.36; p=0.021), indicating that emergency cases constituted the majority of the surgical workload during the study period (Table 1).

**Table 1:** Gender distribution and type of surgery Values are presented as N (frequency) and %. The chi-squared test (χ²) was applied, with significance set at p<0.05.

Variable	Category	N (%)	χ²	P-value
Gender	Male	42 (47.2%)	3.45	0.063
	Female	47 (52.8%)		
Type of surgery	Emergency	60 (67.4%)	5.36	0.021
	Elective	29 (32.6%)		
Total	-	89 (100%)	-	-

Emergency procedures demonstrated SSI rates of 0% in clean wounds, 33.3% in clean-contaminated wounds, 41.4% in contaminated wounds, and 71.9% in dirty wounds. In contrast, elective procedures showed lower infection rates of 7.5%, 14.3%, 20%, and 59.1%, respectively (Table 2).

**Table 2:** Comparison of SSIs between elective and emergency cases across wound classes SSI: surgical site infection

Wound class	Emergency		Elective	
	SSIs/total	SSI rate (%)	SSIs/total	SSI rate (%)
Clean	0/3	0	3/40	7.5
Clean-contaminated	7/21	33.3	5/35	14.3
Contaminated	12/29	41.4	8/40	20
Dirty	41/57	71.9	13/22	59.1
Total	60/110	54.5	29/137	21.2
P-value	0.04		0.03	

The mean age was 39.93±20.81 years (range 19-86), indicating a wide age distribution among participants. The age of study participants who underwent emergency surgeries was 38.5±21.0 years, while the age of those who underwent elective surgeries was 40.7±20.5 years. The difference in age group was statistically non-significant (p=0.55). The average hospital stay was 7.66±3.07 days, reflecting moderate postoperative recovery in most patients. It was significantly higher in those who had emergency procedures (8.1±3.2 days), as compared to those who had elective procedures (6.7±2.5; p=0.034) (Table 3).

**Table 3:** Age of patients and duration of hospital stay Significance was set at p<0.05. N: frequency; SD: standard deviation

Variable	Surgery type	N	Mean	SD	P-value (t-test)
Age (years)	Emergency	60	38.5	21.0	0.55
	Elective	29	40.7	20.5	
Hospital stay (days)	Emergency	60	8.1	3.2	0.034
	Elective	29	6.7	2.5	

Twenty-seven (41.6%) patients presented with one or more comorbidities. The most common comorbidities were diabetes and hypertension (n=20 (22.5%) each), followed by diabetes (n=12 (13.5%)) and hypertension (n=5 (5.6%)). The chi-squared test showed a statistically significant association between comorbidity presence and patient distribution (p<0.5) (Table 4).

**Table 4:** Association of comorbidities among patients with SSIs Values are presented as N and %. The chi-squared test (χ²) was applied for overall association, with significance set at p<0.05. SSIs: surgical site infections

Comorbidity type	N (%)	Valid percent (%)	χ²	P-value
Diabetes	12 (13.5%)	13.5	-	0.042
Hypertension	5 (5.6%)	5.6	-	0.031
Diabetes+ hypertension	20 (22.5%)	22.5	-	0.028
No comorbidities	52 (58.4%)	58.4	-	0.001
Total	89 (100%)	100	8.67	0.042

The overall infection rate was 36%, while the procedure-specific rates varied widely. Laparotomy had the highest SSI rate (38/60; 63.3%), followed by open cholecystectomy (11/20; 55%) and open appendectomy (28/64; 43.8%). Open hernia repair (12/35; 34.3%) and laparoscopic hernia repair (2/12; 16.7%) demonstrated moderate rates, while laparoscopic cholecystectomy (4/31; 12.9%) and laparoscopic appendectomy (3/25; 12%) had the lowest rates. These finding highlights that complex and open abdominal procedures have a higher SSI risk, while minimally invasive surgeries generally show lower rates, contributing to long operative time, contamination, and patient comorbidities (Table 5).

**Table 5:** Procedure-specific rates of SSI SSI: surgical site infection

Procedure	Total surgeries (N)	SSI cases (n)	SSI rate (%)
Laparoscopic appendectomy	25	3	12
Open appendectomy	64	28	43.8
Laparoscopic cholecystectomy	31	4	12.9
Open cholecystectomy	20	11	55
Laparotomy	60	38	63.3
Laparoscopic hernia repair	12	2	16.7
Open hernia repair	35	12	34.3
Total	247	89	36

Identified bacterial isolates

The patients who had SSI had their wound swabs or pus aspirates taken (n=89). Of the 89 bacterial isolates, 50 (56.1%) were *E. coli*, followed by *Klebsiella *species (n=10 (11.23%)), *Pseudomonas aeruginosa *(n=8 (8.9%)), *Enterococcus *(n=6 (6.7%)),* Enterobacter *species (n=6 (6.7%)), *Proteus *species (n=5 (5.61%)), and *Staphylococcus aureus *(n=4 (4.4%)) (Table 6).

**Table 6:** Distribution of bacterial isolates from SSIs among post-abdominal surgery patients Values are presented as N and %. The chi-squared test (χ²) was applied for the overall distribution, with significance set at p<0.05. SSI: surgical site infection

Bacterial isolate	N (%)	Valid percent (%)	Cumulative percent (%)	χ²	p-value
Escherichia coli	50 (56.1%)	56.1	56.1	-	-
*Enterobacter* spp.	6 (6.7%)	6.7	62.8	-	-
Enterococcus faecium	6 (6.7%)	6.7	69.5	-	-
*Klebsiella *species	10 (11.23%)	11.23	80.7	-	-
*Proteus* species	5 (5.61%)	5.61	86.3	-	-
Pseudomonas aeruginosa	8 (8.9%)	8.9	95.6	-	-
Staphylococcus aureus	4 (4.4%)	4.4	100	-	-
Total/overall	89 (100%)	100	-	19.84	<0.05

Antibiotic susceptibility pattern of SSI isolates

In 89 patients, various empirical antibiotics were administered prior to culture results, with ceftriaxone being the most frequently prescribed (41.6%), either alone or in combination with metronidazole (18%). Cefepime plus metronidazole were used in 4.5% of cases, whereas piperacillin-tazobactam was used in 18% of cases. Broad-spectrum carbapenems, including meropenem (9%) and imipenem (9%), were used less commonly and were likely reserved for more severe infections or suspected resistant organisms. Overall, the data indicate a predominant reliance on third-generation cephalosporins, particularly ceftriaxone, as the first-line empirical therapy, with broader agents used selectively.

Antimicrobial susceptibility testing of isolates from SSIs revealed distinct patterns across drug classes. Penicillin had the lowest sensitivity, with only nine (10.1%) isolates sensitive, while resistance was observed in 45 (50.6%), eight (9%) showed no growth, and 27 (30.3%) were intermediate. Cephalosporins also showed high resistance, i.e., 53 (59.6%), while 22 (24.7%) were sensitive, nine (10.1%) showed no growth, and five (5.6%) were intermediate cases. Carbapenems performed comparatively better, with 43 (48.3%) sensitive, 21 (23.6%) resistant, and seven (7.9%) revealing no growth, and 18 (20.2%) were intermediate. Fluoroquinolones showed poor activity, with only 11 (12.4%) sensitive, whereas resistance reached 55 (61.8%). Aminoglycosides demonstrated moderate efficacy, with 44 (49.4%) sensitive, 23 (25.8%) resistant, and six (6.7%) no-growth cases. Macrolides exhibited near-equal proportions of sensitivity, i.e., 43 (48.3%), and resistance, i.e., 40 (44.9%), with no growth in six (6.7%) cases. Among gram-positive agents, vancomycin showed relatively good sensitivity, i.e., 49 (55.1%), five (5.6%) no growth, and 35 (39.3%) intermediate sensitivity, while linezolid retained the highest activity, i.e., 51 (57.3%) sensitive and only three (3.4%) resistant. For β-lactam/β-lactamase inhibitor combinations, 39 (43.8%) were sensitive and 24 (27%) were resistant to piperacillin-tazobactam, while 25 (28.1%) displayed sensitivity and 28 (31.5%) resistance to sulbactam-cefoperazone. Augmentin was the least effective, with only 13 (14.6%) sensitive, 30 (33.7%) resistant, and nearly half of the isolates (n=43 (48.3%)) showing an intermediate response (Table 7).

**Table 7:** Antibiotic susceptibility patterns of bacterial isolates from SSIs Values are presented as N and %, with significance set at p<0.05. SSIs: surgical site infections

Antibiotic/class	Sensitive N (%)	Resistant N (%)	No growth N (%)	Intermediate N (%)	P-value
Penicillin	9 (10.1%)	45 (50.6%)	8 (9%)	27 (30.3%)	-
Cephalosporins	22 (24.7%)	53 (59.6%)	9 (10.1%)	5 (5.6%)	-
Carbapenems	43 (48.3%)	21 (23.6%)	7 (7.9%)	18 (20.2%)	-
Fluoroquinolones	11 (12.4%)	55 (61.8%)	5 (5.6%)	18 (20.2%)	-
Aminoglycosides	44 (49.4%)	23 (25.8%)	6 (6.7%)	16 (18%)	-
Macrolides	43 (48.3%)	40 (44.9%)	6 (6.7%)	0 (0%)	-
Vancomycin	49 (55.1%)	0 (0%)	5 (5.6%)	35 (39.3%)	-
Augmentin	13 (14.6%)	30 (33.7%)	3 (3.4%)	43 (48.3%)	-
Linezolid	51 (57.3%)	3 (3.4%)	5 (5.6%)	30 (33.7%)	-
Piperacillin-tazobactam	39 (43.8%)	24 (27%)	7 (7.9%)	19 (21.3%)	-
Sulbactam-cefoperazone	25 (28.1%)	28 (31.5%)	4 (4.5%)	32 (36%)	<0.05

## Discussion

This study has demonstrated that in patients with SSI following abdominal surgery, gram-negative bacteria predominated with *E. coli *being the most common isolate, i.e., 50 (56.1%), followed by *Klebsiella *species (n=10 (11.23%)) and *Pseudomonas aeruginosa* (n=8 (8.9%)). Antibiotic sensitivity revealed a high resistance to common antibacterial agents like penicillin, cephalosporins, and fluoroquinolones, while carbapenem and linezolid retained higher sensitivity. A major contributor to healthcare expenses, extended hospital stays, and postoperative morbidity, especially in underdeveloped nations, is SSIs [1,2]. Our study aimed to determine which strains of bacteria cause SSI and investigate their sensitivity patterns to antibiotics.

Our study showed that gram-negative organisms dominated 89 SSI patients; the most frequently isolated organism was *E. coli*, i.e., 50 (56.1%), followed by *Klebsiella *species (n=10 (11.23%)) and *Pseudomonas aeruginosa *(n=8 (8.9%)). Gram-positive bacteria were less prevalent, i.e., four (4.4%), especially *Staphylococcus aureus*. These findings are consistent with previous regional studies from Pakistan and other regions such as Bahir Dar, Addis Ababa, Hawassa, and Mekelle, Ethiopia, as well as studies in Nigeria and India, which demonstrated the predominance of gram-negative organisms [20-23]. The predominance of *E. coli *is seen in emergency surgeries, which increases the risk of contamination from endogenous gut flora. Additional contributing factors include comorbidities such as diabetes and hypertension, which compromise host immunity and impair wound healing.

The antimicrobial susceptibility pattern showed substantial resistance to widely used empirical antibiotics, such as fluoroquinolones (n=55 (61.8% resistance)), cephalosporins (n=53 (59.6% resistance)), and penicillin (n=45 (50.6% resistance)). The most efficient antibiotics against gram-negative bacteria were carbapenems (43 (48.3%) sensitivity with 18 (20.2%) intermediate sensitivity), whereas aminoglycosides showed considerable effectiveness (44 (49.4%) sensitivity). Linezolid had the best sensitivity, i.e., 51 (57.3%), among gram-positive cocci, whereas both vancomycin and linezolid showed prolonged activity. In this context, β-lactam/β-lactamase inhibitor combinations, like piperacillin-tazobactam, showed intermediate efficacy, i.e., 39 (43.8% sensitivity), whereas Augmentin showed low activity, i.e., 13 (14.6% sensitivity), suggesting limited usefulness in empirical treatment for SSI [23].

As seen in our study, where ceftriaxone was the most frequently administered antibiotic (41.6%), the significant resistance to third-generation cephalosporins and fluoroquinolones may be related to their extensive empirical use before the culture results. Their preserved activity was likely aided by the selective use of carbapenems and other antibiotics. These results demonstrate the vital necessity of prudent antibiotic management, informed by local susceptibility information, to stop the spread of microbes resistant to multiple drugs [24]. Similar trends have been reported in literature, where overuse of third-generation cephalosporins and fluoroquinolones has led to growing resistance, particularly in low- to middle-income countries.

The likelihood of SSI and the complexity of infections may have been influenced by comorbidities such as diabetes and hypertension, which were present in 37 (41.6%) patients. Furthermore, most operations, i.e., 60 (67.4%), were emergency surgeries, which is in line with the higher infection risks associated with urgent abdominal surgeries that are thought to be caused by contamination, prolonged operating times, and impaired sterility. To prioritize medications with long-term effectiveness and prevent the abuse of broad-spectrum antibiotics, which contribute to resistance, empirical antibiotic regimens must be carefully adjusted to local trends. The critical necessity for institution-specific surveillance of SSI bacteria and their resistance profiles is highlighted by our findings, which are consistent with those of previous studies [25-28]. Furthermore, SSI incidence can be considerably decreased and clinical outcomes enhanced by rigorous adherence to infection control policies, prompt surgical prophylaxis, and management of patient comorbidities.

Clinical implications

Local antibiograms are important to guide empirical antibiotic therapy. Rational antibiotic use can reduce the development of multidrug-resistant infections, hospital stay, and healthcare burden. Carbapenems and linezolid should be reserved for confirmed resistant cases. Additionally, strict infection control, perioperative prophylaxis, and comorbidity management are critical to reduce SSIs.

Future implications

Larger multiple studies are needed to validate these findings across different surgical settings. Research should assess the effectiveness of rapid diagnostic tools for early pathogen identification. Additionally, evaluation of alternative prophylactic regimens and stewardships are warranted to limit antimicrobial resistance.

Limitations

It is a single-centered study with a relatively small sample size. Prior empirical antibiotic use before culture may have altered bacterial isolation and sensitivity results.

## Conclusions

The main cause of SSIs after undergoing abdominal surgery at our facility was gram-negative bacteria, specifically *E. coli*, with a high frequency of multidrug-resistant strains. Linezolid and carbapenems are still good choices, but to reduce antibiotic resistance and enhance surgical results, targeted treatment, cautious antibiotic use, and continuous local surveillance are essential. Future studies should focus on larger, multicenter cohorts, the impact of different surgical prophylaxis protocols, and strategies to prevent multidrug-resistant pathogens.

## References

[1] Nashebi R, Sari M, Kotil SE (2024). Mathematical modelling of antibiotic interaction on evolution of antibiotic resistance: an analytical approach. PeerJ.

[2] Puro V, Coppola N, Frasca A, Gentile I, Luzzaro F, Peghetti A, Sganga G (2022). Pillars for prevention and control of healthcare-associated infections: an Italian expert opinion statement. Antimicrob Resist Infect Control.

[3] (2018). Surgical site infection after gastrointestinal surgery in high-income, middle-income, and low-income countries: a prospective, international, multicentre cohort study. Lancet Infect Dis.

[4] Dessie W, Mulugeta G, Fentaw S, Mihret A, Hassen M, Abebe E (2016). Pattern of bacterial pathogens and their susceptibility isolated from surgical site infections at selected referral hospitals, Addis Ababa, Ethiopia. Int J Microbiol.

[5] Kao LS, Ghaferi AA, Ko CY, Dimick JB (2011). Reliability of superficial surgical site infections as a hospital quality measure. J Am Coll Surg.

[6] Munawar S, Sami A, Rafiq K, Ahmed Z, Shafique A, Ali H, Ahsan S (2024). Analyzing the prevalence and patterns of antibiotic-resistant pathogens causing surgical site infections: antibiotic resistance in surgical site infections. Pak J Health Sci.

[7] Bucataru A, Balasoiu M, Ghenea AE (2023). Factors contributing to surgical site infections: a comprehensive systematic review of etiology and risk factors. Clin Pract.

[8] Hameed S, Hafeez MU, Abdullah W, Faisal UA, Javed A, Iqbal MA (2025). Assessment of surgical site infections in surgeries at tertiary care hospitals in Southern Punjab, Pakistan; a retrospective study. J Popul Ther Clin Pharmacol.

[9] Gillispie-Bell V (2020). Prevention of surgical site infections in gynecologic surgery: a review of risk factors and recommendations. Ochsner J.

[10] Rosenthal VD, Yin R, Jin Z (2024). International Nosocomial Infection Control Consortium (INICC) report of health care-associated infections, data summary of 25 countries for 2014 to 2023, Surgical Site Infections Module. Am J Infect Control.

[11] Klein EY, Milkowska-Shibata M, Tseng KK, Sharland M, Gandra S, Pulcini C, Laxminarayan R (2021). Assessment of WHO antibiotic consumption and access targets in 76 countries, 2000-15: an analysis of pharmaceutical sales data. Lancet Infect Dis.

[12] Klein EY, Van Boeckel TP, Martinez EM (2018). Global increase and geographic convergence in antibiotic consumption between 2000 and 2015. Proc Natl Acad Sci U S A.

[13] Schellack N, Benjamin D, Brink A (2017). A situational analysis of current antimicrobial governance, regulation, and utilization in South Africa. Int J Infect Dis.

[14] Bell BG, Schellevis F, Stobberingh E, Goossens H, Pringle M (2014). A systematic review and meta-analysis of the effects of antibiotic consumption on antibiotic resistance. BMC Infect Dis.

[15] Cassini A, Högberg LD, Plachouras D (2019). Attributable deaths and disability-adjusted life-years caused by infections with antibiotic-resistant bacteria in the EU and the European Economic Area in 2015: a population-level modelling analysis. Lancet Infect Dis.

[16] Vicentini C, Politano G, Corcione S, Furmenti MF, Quattrocolo F, De Rosa FG, Zotti CM (2019). Surgical antimicrobial prophylaxis prescribing practices and impact on infection risk: results from a multicenter surveillance study in Italy (2012-2017). Am J Infect Control.

[17] Bull AL, Worth LJ, Spelman T, Richards MJ (2017). Antibiotic prescribing practices for prevention of surgical site infections in Australia: increased uptake of national guidelines after surveillance and reporting and impact on infection rates. Surg Infect (Larchmt).

[18] Hayat K, Rosenthal M, Gillani AH (2019). Perspective of Pakistani physicians towards hospital antimicrobial stewardship programs: a multisite exploratory qualitative study. Int J Environ Res Public Health.

[19] Iftikhar M, Khan I, Khan SJ, Khan JZ, Rahman SU (2024). Antibiogram and antibiotic resistance patterns in bacterial isolates from Hayatabad Medical Complex, Peshawar. Cureus.

[20] Negi V, Pal S, Juyal D, Sharma MK, Sharma N (2015). Bacteriological profile of surgical site infections and their antibiogram: a study from resource constrained rural setting of Uttarakhand State, India. J Clin Diagn Res.

[21] Admas A, Gelaw B, BelayTessema BelayTessema, Worku A, Melese A (2020). Proportion of bacterial isolates, their antimicrobial susceptibility profile and factors associated with puerperal sepsis among post-partum/aborted women at a referral hospital in Bahir Dar, Northwest Ethiopia. Antimicrob Resist Infect Control.

[22] Ennab R, Al-Momani W, Al-Titi R, Elayan A (2022). Antibiotic profile of pathogenic bacteria isolated from postsurgical site infections in public hospitals in Northern Jordan. Infect Drug Resist.

[23] Bilal H, Khan MN, Rehman T, Hameed MF, Yang X (2021). Antibiotic resistance in Pakistan: a systematic review of past decade. BMC Infect Dis.

[24] Saleem Z, Haseeb A, Abuhussain SS (2023). Antibiotic susceptibility surveillance in the Punjab province of Pakistan: findings and implications. Medicina (Kaunas).

[25] Yayan J, Ghebremedhin B, Rasche K (2015). Antibiotic resistance of Pseudomonas aeruginosa in pneumonia at a single university hospital center in Germany over a 10-year period. PLoS One.

[26] Navon-Venezia S, Kondratyeva K, Carattoli A (2017). Klebsiella pneumoniae: a major worldwide source and shuttle for antibiotic resistance. FEMS Microbiol Rev.

[27] Mangram AJ, Horan TC, Pearson ML, Silver LC, Jarvis WR (1999). Guideline for prevention of surgical site infection, 1999. Am J Infect Control.

[28] Regmi SM, Sharma BK, Lamichhane PP, Gautam G, Pradhan S, Kuwar R (2020). Bacteriological profile and antimicrobial susceptibility patterns of wound infections among adult patients attending Gandaki Medical College Teaching Hospital, Nepal. J Gandaki Med Coll Nepal.

